# The Bacteriophage T4 MotB Protein, a DNA-Binding Protein, Improves Phage Fitness

**DOI:** 10.3390/v10070343

**Published:** 2018-06-26

**Authors:** Jennifer Patterson-West, Melissa Arroyo-Mendoza, Meng-Lun Hsieh, Danielle Harrison, Morgan M. Walker, Leslie Knipling, Deborah M. Hinton

**Affiliations:** Gene Expression and Regulation Section, Laboratory of Cell and Molecular Biology, National Institute of Diabetes and Digestive and Kidney Diseases, National Institutes of Health, Bethesda, MD 20892-0830, USA; jennifer.west@nih.gov (J.P.-W.); melissa.arroyo-mendoza@nih.gov (M.A.-M.); meng-lun.hsieh@nih.gov (M.-L.H.); Dharriso@sgu.edu (D.H.); morgan.walker@yale.edu (M.M.W.); lesliek@mail.nih.gov (L.K.)

**Keywords:** bacteriophage T4, MotB, H-NS, host takeover, DNA-binding protein, bacteriostatic, RNA-seq, transcriptome analysis

## Abstract

The lytic bacteriophage T4 employs multiple phage-encoded early proteins to takeover the *Escherichia coli* host. However, the functions of many of these proteins are not known. In this study, we have characterized the T4 early gene *motB*, located in a dispensable region of the T4 genome. We show that heterologous production of MotB is highly toxic to *E. coli*, resulting in cell death or growth arrest depending on the strain and that the presence of *motB* increases T4 burst size 2-fold. Previous work suggested that *motB* affects middle gene expression, but our transcriptome analyses of T4 *motB^am^* vs. T4 wt infections reveal that only a few late genes are mildly impaired at 5 min post-infection, and expression of early and middle genes is unaffected. We find that MotB is a DNA-binding protein that binds both unmodified host and T4 modified [(glucosylated, hydroxymethylated-5 cytosine, (GHme-C)] DNA with no detectable sequence specificity. Interestingly, MotB copurifies with the host histone-like proteins, H-NS and StpA, either directly or through cobinding to DNA. We show that H-NS also binds modified T4 DNA and speculate that MotB may alter how H-NS interacts with T4 DNA, host DNA, or both, thereby improving the growth of the phage.

## 1. Introduction

An increase in the occurrence of antibiotic-resistant bacteria has sparked an interest in phage–host interactions [1,2]. As bacteriophages have evolved multiple mechanisms to take over their hosts, hosts have responded with mechanisms to thwart takeover. An understanding of these mechanisms would be beneficial to the development of new antibacterial strategies. However, the lack of information about the functions of a large portion of phage genes [3] has hampered this investigation.

The lytic bacteriophage T4 infects *Escherichia coli*, resulting in rapid cell lysis after ~20 min at 37 °C. Despite the use of T4 as a model organism for decades, the functions of many genes, especially those expressed early during infection, remain unknown [4]. As T4 does not encode its own RNA polymerase (RNAP), it must use the host’s RNAP to program its temporal pattern of early, middle, and late gene expression (reviewed in [5]). T4 directs RNAP to early, middle and late promoters by encoding factors that change the specificity of RNAP as infection proceeds. Early RNAs, which are expressed immediately after infection, arise from T4 early promoters (Pe’s). Pe’s, whose activation does not require phage-encoded factors, contain a very strong match to the consensus sequences of host promoters, allowing them to compete with host DNA for the same pool of RNAP. T4 middle transcripts are expressed approximately 1 to 2 min post-infection at 37 °C from middle promoters (Pm’s) and through the extension of early transcription into downstream middle genes. Activation of Pm’s requires two T4 proteins that modify RNAP, MotA and AsiA, resulting in the recognition of promoters that have a new DNA motif [6]. T4 late transcripts, which are expressed ~5 min after infection, use late promoters (Pl’s) that contain a novel −10 sequence known as the TATA box. The T4-encoded late σ factor, σ^55^ (gene product [gp]55), recognizes this element, facilitating late transcription along with the coactivator, gp33, and the sliding clamp, gp45 (reviewed in [7]).

The histone-like protein H-NS and its less abundant homolog StpA are known to protect bacteria from the expression of foreign genes. H-NS is a DNA-binding protein that targets AT-rich DNA sequences, condensing genomic DNA through the formation of ordered and looped structures that typically repress transcription at the affected region (reviewed in [8]). As phage genomes and xenogeneic sequences acquired from horizontal gene transfer often display a high AT content, H-NS can repress expression of this DNA (reviewed in [9]). Given the AT-richness of T4 DNA (65.5%), it would not be surprising for H-NS binding to exert such an inhibitory effect on T4 gene expression. In fact, previous work has shown that the T4 Arn protein, an early gene product that structurally mimics DNA, can bind H-NS, preventing its interaction with DNA and formation of higher order structures [10]. However, Arn is not essential [11] and whether its absence affects T4 gene expression is not known. Specific H-NS antagonists have also been identified for T7 [12], Luz24 [13], and Mu [14,15].

Like *arn*, *motB* is an early gene that is not essential. It is located in a dispensable region of the T4 genome [16], where a subset of genes have been shown to modulate gene expression and/or host functions under certain growth conditions [17]. For instance, *mrh* and *srh* affect late T4 transcription under heat shock (42 °C) and in certain *rpoH* (σ^32^) mutants. In addition, *69*, *modA*, and *srd* impair *E. coli* growth when heterologously expressed [17]. Previously, *motB* has been implicated in the optimal expression of certain T4 middle genes [16] and was given the name *motB* for modifier of transcription B. In addition, MotB protein, identified as p17.6, was isolated in a prereplicative complex containing DNA, RNAP, MotA, AsiA, and other polymerase-associated and DNA-binding proteins [18]. However, its function has remained unknown.

We show here that the presence of *motB* increases T4 burst size twofold, and heterologous production of MotB is highly toxic to *E. coli*, resulting in cell death or growth arrest depending on the strain. Our transcriptome analyses of T4 wild type (wt) RNA vs. T4 *motB^am^* RNA reveal only mild impairment of a few late genes at 5 min post-infection, while early and middle genes are not significantly affected. Consequently, it seems unlikely that MotB is directly involved in T4 gene expression. We have purified MotB and demonstrated that it binds to unmodified and modified [(glucosylated, hydroxymethylated-5 cytosine, (GHme-C)] DNA fragments with no detectable sequence specificity. Interestingly, MotB copurifies with the host histone-like proteins, H-NS and StpA, either directly or through cobinding to DNA, and we find that H-NS also binds T4 modified DNA. We speculate that MotB may alter how H-NS interacts with host DNA, T4 DNA, or both, thereby improving T4 growth.

## 2. Materials and Methods

### 2.1. DNA

pTE103-*motB^am^* was prepared by cloning the 489 base pair (bp) sequence preceding and following the codon for *motB* residue S12, mutated from TCT to TAG, between the BamHI and SalI sites. pNW129-MotB was constructed by cloning *motB*, whose sequence was optimized for codon usage, between KpnI and SalI sites of the kan^r^, pACYC-derived vector pNW129 [19]. In this arrangement, *motB* is appropriately downstream of a ribosome-binding site and is under the control of the arabinose inducible promoter P*_BAD_*. pNW129-MotB-His was constructed as described for pNW129-MotB except the native stop codon was omitted so that the C-terminal His_6_-tag was transcribed. pTXB1-MotB was obtained by cloning the native sequence minus its stop codon between the NdeI and SapI sites of pTXB1 (New England BioLabs, Ipswich, MA, USA). The resulting plasmid produces MotB fused to an auto-cleavable C-terminal intein with a chitin-binding domain (CBD) under the control of the isopropyl β-d-thiogalactopyranoside (IPTG) inducible T7 promoter. pTXB1-HNS was constructed as described for pTXB1-MotB. GenScript (Piscataway Township, NJ, USA) performed gene synthesis, plasmid construction, and DNA sequencing for the plasmids used in this study.

T4 DNA was purified from phage extraction with phenol, phenol:chloroform:isoamyl alcohol (25:24:1), and chloroform:isoamyl alcohol (24:1) (two extractions with each solvent). DNA was then dialyzed into 10 mM Tris-HCl (pH 8.0) and 1 mM ethylenediaminetetraacetic acid (EDTA) at 4 °C. λ DNA was purchased from New England BioLabs. Where indicated, T4 and λ DNA were treated with SspI (New England BioLabs) and HindIII (New England BioLabs), respectively, purified by phenol extraction/ethanol precipitation, and dissolved in nuclease-free water.

Oligonucleotides used for primer extensions, RT-qPCR, and gel retardation assays were synthesized by Integrated DNA Technologies (Coralville, IA, USA); sequences are available upon request. Radiolabeled oligonucleotides were prepared by treating the top strand oligonucleotide with OptiKinase (Affymetrix, Santa Clara, CA, USA) in the presence of [γ-^32^P]ATP. ^32^P-labeled single stranded (ss) DNA, purified by phenol extraction/butanol precipitation, was resuspended in 1× TE (Quality Biological, Gaithersburg, MD, USA). Double-stranded (ds) oligonucleotides were prepared by mixing the ^32^P-labeled top strand with the complementary bottom strand in 1× OptiKinase Reaction Buffer (Affymetrix, Santa Clara, CA, USA), heating at 92 °C for 2 min, and slowly cooling to room temperature. The ^32^P-labeled dsDNA was subsequently purified using a G-25 microspin column (GE Healthcare, Little Chalfont, UK).

The ^32^P-labeled PCR product for DNase I footprinting was obtained using Pfu Turbo polymerase (Stratagene, San Diego, CA, USA), upstream and downstream primers that annealed from positions −143 to +75 of the T4 late promoter for *gp8* (Pl*_8_*), and purified T4 DNA. The top strand (nontemplate) primer was treated with T4 polynucleotide kinase (Affymetrix) in the presence of [γ-^32^P]ATP prior to PCR. The PCR product was isolated after gel electrophoresis using an Elutrap (GE Healthcare) and ethanol precipitated.

### 2.2. Bacterial and Bacteriophage Strains

*E. coli* strains TOP10F’ (Invitrogen, Carlsbad, CA, USA), BL21(DE3), and BL21(DE3)/pLysE [20] were used for expression studies. NapIV suppressing (NapIV S) and NapIV wt (non-suppressing, NapIV NS) [21] *E. coli* were used for T4 infections. Unless otherwise noted, cells were grown at 37 °C with shaking at 250 rpm.

Wild-type T4D^+^ (T4 wt), T4 *amG1* (T4 *motA^am^*) [22], and T4 *motB^am^* were used for infections. T4 *motB^am^* was obtained by recombination of pTE103-*motB^am^* into the T4 genome. BL21(DE3)/pTE103-*motB^am^* was infected with T4 wt at a multiplicity of infection (MOI) of 1 during exponential phase and incubated for 75 min. Cells were lysed with chloroform and resulting phage were titered on NapIV S. Plaques were screened for the presence of the amber mutation by hybridization of a ^32^P-labeled probe containing either the wt or mutant sequence as described [23]. Potential mutant plaques were used to generate phage stocks by infecting NapIV S. Phage stocks containing the amber mutation were subjected to a subsequent round of single plaque selection to ensure a homogenous stock of T4 *motB^am^*. To confirm the presence of the amber mutation, the *motB* gene was amplified by PCR then sequenced by Macrogen (Rockville, MD, USA).

### 2.3. MotB Toxicity Assay

BL21(DE3) and TOP10F’ containing pNW129 (empty vector) or pNW129-MotB were plated on 1.5% (*w*/*v*) LB agar (Quality Biological or Sigma, St. Louis, MO, USA) containing 40 µg/mL kanamycin, 12 µg/mL tetracycline (TOP10F’ only), and 0.5% (*w*/*v*) glucose. Overnight cultures were grown in LB (Quality Biological) containing 40 µg/mL kanamycin, 12 µg/mL tetracycline (TOP10F’ only), and 0.025% (*w*/*v*) glucose. Overnight cultures were diluted to an OD_600_ of 0.1 with LB containing 40 µg/mL kanamycin and 12 µg/mL tetracycline (TOP10F’ only). At an OD_600_ of approximately 0.3, 0.2% (*w*/*v*) arabinose (final concentration) was added to each sample. At the indicated times samples were taken and electrophoresed on SDS-PAGE gels to monitor MotB production.

### 2.4. Protein Purification

#### 2.4.1. MotB Purification (Method I)

MotB containing a C-terminal intein tag with a chitin binding domain (MotB-Intein/CBD) was isolated and purified from BL21(DE3)/pLysE containing pTXB1-MotB. Overnight cultures were grown in LB containing 25 µg/mL chloramphenicol and 100 µg/mL carbenicillin. Cells were diluted to an OD_600_ ~0.1 in LB containing 25 µg/mL chloramphenicol and 100 µg/mL carbenicillin and then grown at 25 °C with shaking at 250 rpm. At an OD_600_ between 0.3 and 0.4, synthesis of MotB-Intein/CBD was induced by the addition of 4 mM IPTG (final concentration) for 2 h. Cells were harvested by centrifugation at 13,000× *g* for 10 min and stored at −80 °C. Unless otherwise noted, the following procedures were performed on ice or at 4 °C. Cells were resuspended in CB Buffer (20 mM HEPES-OH (pH 8.5), 50 mM NaCl, 1 mM EDTA, 0.01% (*v*/*v*) Triton X-100) containing 1 mM benzamidine, then lysed by sonication until OD_600_ was reduced ~3-fold. Clarified supernatant was obtained by centrifugation at 15,000× *g* for 30 min followed by filtration through a 0.4 µm syringe filter. Chitin resin (New England BioLabs) equilibrated with CB Buffer (5 mL slurry per 125 mL starting culture) was added to the supernatant, and the suspension was gently rocked overnight. Resin was transferred to a 30 mL disposable column (Bio-Rad, Hercules, CA, USA) and then washed with ~25 column volumes of CB Buffer containing 1 M NaCl at a flow rate of <2 mL/min (gravity drip) until the A_260_ reached a minimum. The resin was subsequently washed with an additional 7 column volumes of CB Buffer containing 500 mM NaCl. On-column cleavage of the Intein/CBD-tag was initiated by quickly washing the column with 3 column volumes of CB Buffer containing 500 mM NaCl and 1 mM dithiothreitol (DTT). After ≥40 h, MotB was eluted by the addition of 2 column volumes of CB Buffer containing 500 mM NaCl and 2 mL fractions were collected. Fractions containing MotB were identified by SDS-PAGE and then dialyzed into Buffer A (25 mM Tris-HCl (pH 8.0), 50 mM NaCl, 6 M deionized Urea). The denatured MotB was isolated after passage through a 1 mL HiTrap Q column (GE healthcare), in which MotB was in the flow-through fraction while residual nucleic acid bound to the column. MotB was then refolded using the dialysis procedure previously described for *E. coli* σ^70^ renaturation [24] and stored at −20 °C in MotB storage buffer (50 mM Tris-HCl (pH 8.0), 1 mM EDTA, 50 mM NaCl, 0.01% *v*/*v* Triton X-100, 50% *v*/*v* glycerol, 0.1 mM DTT).

#### 2.4.2. MotB Purification (Method II)

MotB containing a C-terminal His_6_-tag (MotB-His) was isolated and purified from TOP10F’ containing pNW129-MotB-His. Overnight cultures were grown in LB containing 12 µg/mL tetracycline, 40 µg/mL kanamycin, and 0.05% (*w*/*v*) glucose. Cells were diluted to an OD_600_ ~0.1 in LB containing 12 µg/mL tetracycline and 40 µg/mL kanamycin and grown to an OD_600_ around 0.3, when synthesis of MotB-His was induced by the addition of 0.2% (*w*/*v*) arabinose (final concentration) for 1 h. Cells were harvested by centrifugation at 13,000× *g* for 10 min and stored at −80 °C. Unless otherwise noted, the following procedures were performed on ice or at 4 °C. Cells were resuspended in 40 mL of Lysis Buffer (25 mM Tris-HCl (pH 8.0), 50 mM NaCl, 5 mM imidazole, 0.01% (*v*/*v*) Triton X-100, 1 mM benzamidine) per 1 L culture, lysed by sonication as described above and then centrifuged at 17,500× *g* for 20 min. The pellet, containing MotB-His, was resuspended in Lysis Buffer and centrifuged again at 17,500× *g* for 15 min. To recover MotB-His, the pellet was resuspended in 20 mL Binding Buffer (20 mM Tris-HCl (pH 8.0), 500 mM NaCl, 5 mM imidazole, 6 M urea, 1 mM benzamidine, pH adjusted to 7.9) per 1 L culture, incubated for 1 h with gentle mixing, and then centrifuged at 17,500× *g* for 15 min. The supernatant, containing MotB-His, was then incubated with gentle mixing for a minimum of 1 h with 5 mL (per 1 L culture) Ni-NTA His-binding resin (Millipore, Burlington, MA, USA) that was pre-equilibrated with Binding Buffer. Resin was transferred to a 30 mL disposable column (Bio-Rad) then washed with four column volumes of Binding Buffer followed by six column volumes of Wash Buffer (25 mM Tris-HCl (pH 8.0), 500 mM NaCl, 60 mM imidazole, 6 M urea, pH adjusted to 7.9) at a flow rate of <2 mL/min (gravity drip). The major fraction of MotB-His eluted with 2 column volumes of Elution Buffer 1 (20 mM Tris-HCl (pH 8.0), 500 mM NaCl, 200 mM imidazole, 6 M urea, pH adjusted to 7.9). Residual MotB-His was subsequently eluted with 2 column volumes of Elution Buffer 2 (20 mM Tris-HCl (pH 8.0), 500 mM NaCl, 400 mM imidazole, 6 M urea, pH adjusted to 7.9). For both elution conditions, 2.5 mL fractions were collected. Fractions containing MotB-His, identified by SDS-PAGE, were refolded as described in Method I.

#### 2.4.3. H-NS Purification

H-NS containing a C-terminal intein tag with a chitin binding domain (H-NS-Intein/CBD) was isolated and purified from BL21(DE3)/pLysE containing pTXB1-H-NS. Overnight cultures were grown in LB containing 25 µg/mL chloramphenicol and 100 µg/mL carbenicillin. Cells were diluted to an OD_600_ ~0.1 in LB containing 25 µg/mL chloramphenicol and 100 µg/mL carbenicillin and then grown at 25 °C with shaking at 250 rpm. At an OD_600_ between 0.3 and 0.4, synthesis of H-NS-Intein/CBD was induced by the addition of 4 mM IPTG (final concentration) for 2 h. Cells were harvested by centrifugation at 13,000× *g* for 10 min and stored at −80 °C. Unless otherwise noted, the following procedures were performed on ice or at 4 °C. Cells were resuspended in 30 mL of H-NS Sonication Buffer (20 mM Tris-HCl (pH 8.0), 750 mM NaCl, 1 mM EDTA, 0.01% (*v*/*v*) Triton X-100, 1 mM Benzamidine) then lysed by sonication until OD_600_ was reduced ~3-fold. Clarified supernatant was obtained by centrifugation at 15,000× *g* for 30 min followed by filtration through a 0.45 μm syringe filter. Chitin resin (New England BioLabs) equilibrated with H-NS Sonication Buffer (5 mL slurry per 250 mL starting culture) was added to the supernatant and rocked overnight. Resin/cell lysate was transferred to a 30 mL disposable column (Bio-Rad) and washed with approximately 20 column volumes of CCB buffer (20 mM Tris-HCl (pH 8.0), 500 mM NaCl, 1 mM EDTA, 0.01% (*v*/*v*) Triton X-100) at a flow rate of <2 mL/min (gravity drip). On-column cleavage of the Intein/CBD-tag was initiated by quickly washing the column with 3 column volumes of CCB buffer containing 50 mM DTT. After ≥40 h, H-NS was eluted by the addition of 3 column volumes of CCB buffer and 1 mL fractions were collected. Fractions containing H-NS were identified by SDS-PAGE and pooled. As this pool contained a small amount of intein tagged protein, it was passed over a new column containing 5 mL of fresh, equilibrated chitin slurry, and fractions from the flow-through plus 2 column volume washes were collected. The now pure H-NS was dialyzed into H-NS Storage Buffer (10 mM potassium phosphate (pH 7.5), 200 mM NaCl, 0.1 mM EDTA, 50% (*v*/*v*) glycerol) and stored at −20 °C.

### 2.5. Identification of Nucleic Acid Copurifying with MotB

MotB samples used to identify copurifying nucleic acid were purified using MotB purification Method I with the following modifications. Elution fractions from the chitin column that contained MotB as determined by SDS-PAGE were directly dialyzed into MotB Storage Buffer and stored at −80 °C. Protein was removed by chloroform:isoamyl alcohol (24:1) extraction, and sodium acetate and glycogen (Thermo Scientific, Waltham, MA, USA) were added to the aqueous phase at a final concentration of 496 mM and 0.17 mg/mL, respectively. The nucleic acid was then ethanol precipitated, resuspended in nuclease-free water, and quantified by NanoDrop 2000c (Thermo Scientific).

To identify nucleic acid, 1× DNase buffer (Ambion, Carlsbad, CA, USA) was added to ~300–400 ng nucleic acid followed by the addition of either 2 units of RNase-free DNase I (Ambion) or 100 µg of DNase-free RNase A and incubation at 37 °C for 10 min. Treated samples and the untreated control were electrophoresed on 0.8% (*w*/*v*) agarose gels at 120 V for 45 min in 1× TAE (Quality Biological) at room temperature. Gels were stained with ethidium bromide for 1 min and visualized using UV illumination. Two independent replicates were performed.

### 2.6. Electrophoretic Mobility Shift Assays (EMSAs)

EMSAs with oligonucleotides (10 µL) contained the indicated amount of MotB, 0.01 pmol of ^32^P-labeled DNA, 40 mM Tris acetate (pH 7.9), 20 mM Tris-HCl (pH 8.0), 2 mM potassium phosphate (pH 7.5), 60 mM NaCl, 150 mM potassium glutamate, 4 mM magnesium acetate, 0.52 mM EDTA, 0.14 mM DTT, 0.1 mg/mL Bovine Serum Albumin (BSA), 0.004% (*v*/*v*) Triton X-100, and 30% (*v*/*v*) glycerol. In the case of the oligonucleotide ProU-40, the solution contained 40 mM Tris acetate (pH 7.9), 20 mM Tris-HCl (pH 8.0), 20 mM Tris-HCl (pH 7.6), 70 mM NaCl, 150 mM potassium glutamate, 4 mM magnesium acetate, 2 mM magnesium chloride, 0.5 mM EDTA, 0.14 mM DTT, 0.1 mg/mL BSA, 0.004% (*v*/*v*) Triton X-100, and 20% (*v*/*v*) glycerol. Solutions were incubated for 10 min at 37 °C, collected on ice, and electrophoresed on 12% (*w*/*v*) polyacrylamide (37.5:1, acrylamide:bis) gels in 1× TBE at 150 V for 3 h at room temperature. Gels were visualized by autoradiography, and the films were scanned using a GS-800 calibrated densitometer (Bio-Rad). Quantification was performed using Quantity One software (Bio-Rad). *K*_*d*(*app*)_ was determine as the protein concentration needed to shift 50% of the ^32^P-labeled DNA.

For gel shift assays with T4 or λ DNA, 60 pmol MotB-His and/or H-NS were incubated with 500 ng of either T4 DNA treated with SspI or λ DNA treated with HindIII in a reaction mixture (5 µL) containing 20 mM Tris-HCl (pH 8), 2 mM potassium phosphate (pH 7.5), 60 mM NaCl, 0.42 mM EDTA, 0.04 mM DTT, 0.004% (*v*/*v*) Triton X-100, and 30% (*v*/*v*) glycerol at 37 °C for 10 min. Samples were electrophoresed on 0.8% (*w*/*v*) agarose gels at 70 V for 90 min in 1× TAE at 4 °C. Gels were stained with ethidium bromide for 1 min and visualized using UV illumination.

### 2.7. DNase I Footprinting

Solutions were assembled in a total volume of 10 µL using 0.005 μM DNA (1 μL) and 0 to 3.2 μM of MotB (4 μL), as indicated, in the same buffer used for gel shift assays with oligonucleotides as described above. After 10 min of incubation at 37 °C, 0.8 units of DNase I (1.1 μL) was added to the reaction. Solutions were incubated for an additional 45 s at 37 °C and quenched with equal volumes of phenol. DNA was extracted, ethanol precipitated, and electrophoresed on 5% (*w*/*v*) polyacrylamide (19:1, acrylamide:bis), 7 M urea denaturing gels for 2500 volt-h in 0.5× TBE. After electrophoresis, gels were visualized by autoradiography and analyzed as described above.

### 2.8. Pull-Down Assay

BL21(DE3)/pLysE containing pTXB1-MotB or pTXB1 was grown in LB containing 25 μg/mL chloramphenicol and 100 μg/mL carbenicillin at 25 °C. MotB was isolated as described in Method I except that after binding to the chitin column, the resin was washed with 5 column volumes of CB buffer containing 500 mM NaCl followed by 5 column volumes of CB buffer containing 1 M NaCl. Proteins, isolated from the SDS-PAGE gel, were analyzed by mass spectrometry (Whitehead Institute, Cambridge, MA, USA).

### 2.9. Bacteriophage Fitness Assays

Overnight cultures were diluted in fresh LB to an OD_600_ ~0.1, grown to early log phase (OD_600_ ~0.3), and then transferred to ice until ready to titer. For each titer, ~50–100 phages in 100 μL LB were incubated with 200 μL of culture for 5 min at room temperature. Following addition of 2.5 mL 0.65% (*w*/*v*) Drake top agar at 45 °C [25], cells were plated on 1.5% (*w*/*v*) LB agar (Sigma) and incubated at 37 °C overnight. For infections using cells containing pNW129-MotB or pNW129, cultures were grown to an OD_600_ ~0.4 as described above and then protein synthesis was induced by the addition of 0.1% (*w*/*v*) arabinose (final concentration) for 20 min before collecting on ice.

Burst size experiments were performed as described [26] for single-step growth experiments with the following modifications. NapIV S and NapIV NS were grown to mid log phase (OD_600_ ~0.5). The NapIV S culture was transferred to ice for use as the plating strain. NapIV NS (900 μL) was transferred to a 1.5 microcentrifuge tube containing 100 μL of phage (MOI of ~0.004) pre-warmed at 37 °C. At 10 min post-infection, the phage-culture sample was diluted 10,000-fold in LB warmed to 37 °C with gentle vortexing to mix and returned to 37 °C. For pre-burst (≤30 min) and post-burst (35 to 70 min) time points, 100 μL or 10 μL of the diluted phage culture, respectively, were added to 100 μL of NapIV S followed by the addition of 3 mL of 0.65% Drake top agar (at 50 °C). The cells were immediately plated on 1.5% (*w*/*v*) LB agar and incubated overnight at 37 °C. Burst size was calculated by dividing the average number of plaques for the post-burst time points (50 to 70 min) by the average number of plaques for pre-burst time points (15 to 30 min).

### 2.10. Purification of Total RNA

NapIV NS were grown to a cell density of ~4 × 10^8^ cells/mL (OD_600_ ~0.4) and then infected with either T4 wt or T4 *motB^am^* at a MOI of 10. As indicated, RNA was isolated at 1, 5 or 10 min post-infection using method II as described [27]. Briefly, cells were chemically lysed and nucleic acid was isolated by hot phenol extraction followed by ethanol precipitation. Samples were then treated with DNase, and total RNA was isolated by phenol extraction followed by ethanol precipitation. Total RNA used for RNA-seq was run on a Bioanalyzer using the Agilent RNA 6000 Nano Kit (Agilent Technologies, Santa Clara, CA, USA) to evaluate the sample quality [28]. RNA used for RT-qPCR and primer extensions was evaluated using a 0.8% (*w*/*v*) agarose gel stained with ethidium bromide.

### 2.11. Transcriptome Analysis

rRNA subtraction was performed with the bacterial RiboMinus Kit (Ambion) according to manufacturer instructions. cDNA was prepared using the NEBNext strand specific kit (New England BioLabs) according to manufacturer instructions for libraries with 300–450 bp insert size with the following modifications. Illumina adaptor sequences based on TruSeq HT Sample Prep Kits were purchased from Integrated DNA Technologies and used in the ligation step. TruSeq-1 and TruSeq-2 primer were used for PCR enrichment of adaptor ligated DNA. Library size was verified with a Bioanalyzer using an Agilent High Sensitivity DNA kit (Agilent Technologies). The concentration of each library was determined using the KAPA Library Quantification Kit (Roche, Basel, Switzerland) for Illumina platforms. Sequencing was performed by the NIDDK Genomics Core facility using a MiSeq system with the MiSeq 2 × 250 bp Sequencing Kit (Illumina, San Diego, CA, USA).

Quality trimming (max ambiguity = 2) and trimming of 15 nucleotides from the 5’ terminal of all sequences were performed with CLC Genomics Workbench (version 8.5, Qiagen, Hilden, Germany). The quality of raw sequencing data was accessed in CLC Biomedical Workbench software (Qiagen). A bimodal distribution of GC-content was observed in the data set with peak maximums at 36% GC and 55% GC (Figure S1). This distribution is consistent with the presence of both host-encoded (~55% GC) and T4-encoded (34.5% GC) transcripts within the data set.

Trimmed data were aligned to the *E. coli* reference genome (NC_012971.2) and an unmapped reads file was generated. Approximately 44–54% of total reads mapped to the host genome. A significant number of host-encoded transcripts is consistent with other transcriptome analysis of bacteriophage infections during early and middle stages of temporal expression [29,30]. The unmapped reads file, presumably containing sequences derived from the T4 genome, was subsequently aligned to the bacteriophage T4 reference genome (NC_000866.4). To mitigate the occurrence of multi-mapping reads, overlapping annotations of genes with alternative start sites or introns were resolved to a single annotation in the T4 reference genome to enable unique gene assignment by CLC Biomedical Workbench software. Differential expression experiments consisted of 3 replicates of NapIV NS infected with either T4 wt or T4 *motB^am^*. Statistical analysis was performed in CLC Genomics Workbench using the Baggerley’s Test with False Discovery Rate (FDR) corrected *p* values. RNA-seq data is available in the NCBI database (GEO number GSE113425) and in Table S1.

### 2.12. RT-qPCR

RNA was reverse transcribed into cDNA using the iScript cDNA Synthesis Kit (BioRad), and target genes were amplified with iTaq Universal SYBR green supermix (BioRad) in a BioRad CFX95. The relative gene expression was determined with the comparative *C*_t_ method using CFX manager software (Bio-Rad), also known as 2^−ΔΔ*C*t^ [31]. Genes *frd.2*, *nrdD,* and *motA,* whose expression was unaffected in RNA-seq analysis, were used as endogenous controls. Primers were designed using Primer3web [32,33]; sequences are available upon request.

### 2.13. Primer Extension

Primer extension was performed using Avian Myeloblastosis Virus (AMV) reverse transcriptase (Life Sciences, Inc., St. Petersburg, FL, USA) as previously described [34,35,36]. Oligonucleotides were designed to anneal ~150 nucleotides (nt) downstream of the predicted 5’ end unless otherwise stated; primer sequences are available upon request. Total RNA (4 μg) was added to 1 pmol of 5’ ^32^P-labeled primer for each analysis. Primer extension products were separated on 5% (*w*/*v*) denaturing, polyacrylamide (19:1, acrylamide:bis) gels. Gels were visualized by autoradiography and analyzed as described above.

## 3. Results

### 3.1. MotB is Toxic when Expressed in E. coli

**Figure 1:**
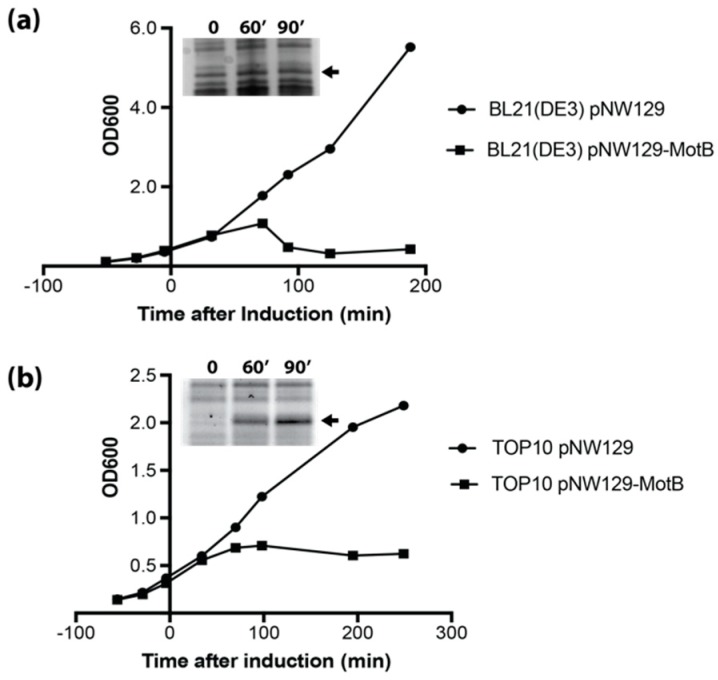
MotB is toxic to *E. coli.* Heterologous expression of *motB* in (**a**) BL21(DE3) or (**b**) TOP10F’ *E. coli* causes cell lysis or inhibits growth, respectively. Cell lines containing either the empty vector pNW129 (circles) or expression vector pNW129-MotB (squares) were induced with 0.2% (*w*/*v*) arabinose (final concentration) at an OD_600_ between 0.3 and 0.4. Growth curves are representative of three independent replicates. To monitor the amount of MotB produced, cell aliquots at 0, 60 and 90 min post-induction were analyzed by SDS-PAGE. The protein gel slice containing MotB is shown for a representative experiment with the band corresponding to MotB designated by a black arrow. It should be noted that although MotB production is seen at 60 min for BL21(DE3)/pNW129-MotB, no additional accumulation is apparent at 90 min perhaps because of the significant cell death at this time point.

Initial efforts to heterologously express *motB*, which had been cloned downstream of the arabinose-inducible promoter P*_BAD_*, in the *E. coli* B strain derivative BL21(DE3) were prevented by plasmid mutation(s) that eliminated the expression of *motB*. To mitigate this issue, all overnight cultures included low levels of glucose to reduce leaky expression from the P*_BAD_* promoter. To assess MotB toxicity, induction of MotB synthesis was done in both BL21(DE3) and in the *E. coli* K12 strain derivative TOP10F’ by the addition of arabinose. In BL21(DE3), a rapid reduction in cell density, as monitored by the OD_600_, was observed approximately 90 min after induction (Figure 1a), indicating cell lysis. At 60 min, a small amount of induced protein that comigrated as MotB was detectible by SDS-PAGE (Figure 1a inset). MotB production in TOP10F’ led to inhibition of cell growth around 60 min after induction without evidence of cell lysis (Figure 1b). For TOP10F’ cells, a significant amount of MotB was produced at 60 min post-induction, indicating that the difference in toxicity was not due to a decrease in expression (Figure 1b inset). Genome-wide comparisons of the transcriptomes and proteomes of *E. coli* B and K-12 strains have shown extensive differences, which could account for the difference in MotB toxicity between the cell lines [37].

### 3.2. MotB Copurifies with DNA

**Figure 2:**
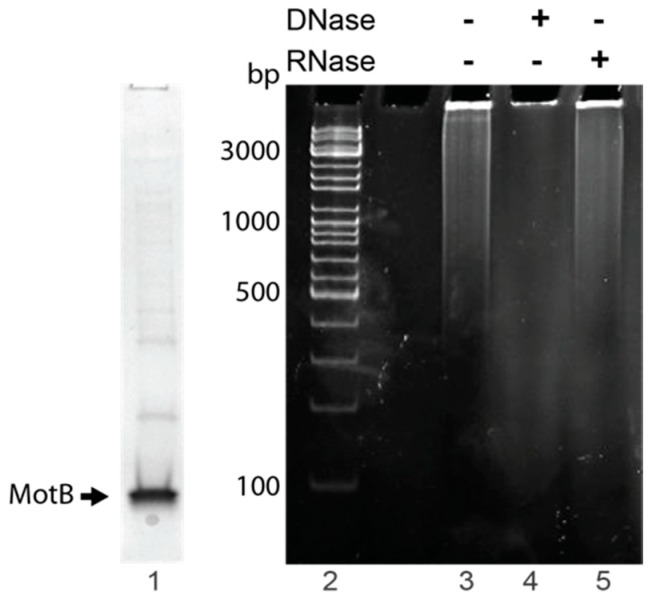
MotB copurifies with host DNA when heterologously produced in *E. coli*. Lane 1 shows an SDS-PAGE gel, stained with Coomassie, indicating the MotB protein used for the nucleic acid extraction. The agarose gel on the right shows copurifying nucleic acid (lane 3) isolated after phenol-chloroform extraction/ethanol precipitation of affinity-purified MotB and then treated with either RNase-free DNase I (lane 4) or DNase-free RNase A (lane 5). Lane 2 contains GeneRuler DNA marker (Invitrogen). Results are representative of two independent samples.

MotB is highly basic with a predicted pKa of 9.23, suggesting that it might interact with nucleic acid. Furthermore, the protein (identified as p17.6) was previously found as part of a prereplicative DNA complex after T4 infection [18], again suggesting a possible interaction with DNA. Our first attempts to purify MotB were thwarted by high levels of nucleic acid that remained with the protein throughout purification. Consequently, we constructed a plasmid that produced MotB with an auto-cleavable C-terminal Intein/CBD tag in the hope that extensive salt washes while the protein was bound to the column would remove DNA/RNA. To monitor the level of nucleic acid during purifications, we measured the 260/280 nm absorbance ratio [38]. Highly purified MotB was obtained after absorption/elution from a chitin column (Figure 2, lane 1). However, even though the resin was washed extensively with buffer containing 1 M NaCl before protein elution, the purified fraction had a 260/280 ratio of between 1.21 and 1.35. To isolate the copurifying nucleic acid, an aliquot of the fraction was extracted with phenol and precipitated with ethanol (Figure 2, lane 3). The resulting nucleic acid was degraded after treatment with DNase (Figure 2, lane 4) but resistant to RNase (Figure 2, lane 5), indicating that the copurifying nucleic acid was DNA.

To remove the DNA, MotB was purified under denaturing conditions using either the MotB-Intein/CBD or a MotB-His construct and then refolded, giving a final 260/280 nm ratio of ~0.43–0.53, consistent with the loss of DNA. Removal of DNA was further confirmed by native gel electrophoresis with ethidium bromide staining. MotB-Intein/CBD was considerably less toxic (Figure S2a) than wt MotB when expressed in *E. coli*, whereas MotB-His retained comparable toxicity (Figure S2b). However, it is important to note that the reduced toxicity of the MotB-Intein/CBD could also be due to growth and induction at 25 °C instead of 37 °C. The culture was grown at 25 °C to improve folding and solubility of the fusion protein as recommended by the IMPACT kit instruction manual (New England BioLabs). Both proteins were highly purified (Figure S3a and S3b). Subsequent assays detailed below indicate that both the refolded His_6_-tagged MotB and the refolded wt MotB generated after intein cleavage have similar and strong DNA-binding activity.

### 3.3. MotB Binds DNA with No Detectable Sequence Specificity

**Figure 3:**
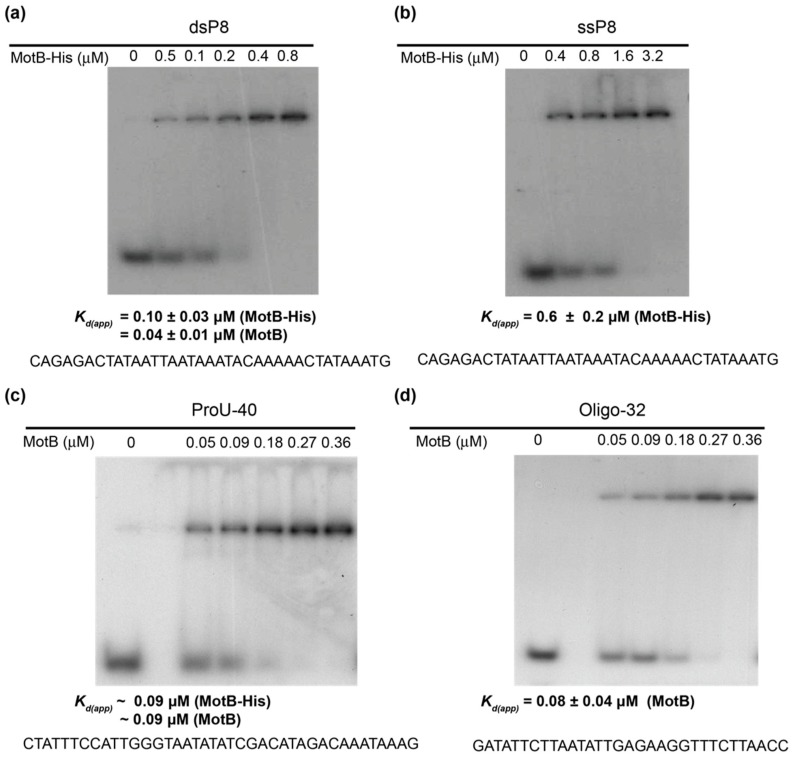
MotB binds ds and ssDNA. Representative EMSAs for MotB and MotB-His binding to (**a**) 37 bp P8 oligonucleotide, which contains the T4 Pl*_8_* promoter from −29 to +8 (dsP8); (**b**) the top strand of the P8 oligonucleotide (ssP8); (**c**) a 40 bp fragment containing the *E. coli proU* operon from +8 to +47 (ProU-40); and (**d**) a random 32 bp oligonucleotide (Oligo-32). DNA (1 nM) was incubated with the indicated concentrations of MotB or MotB-His at 37 °C for 10 min before electrophoresis on a native polyacrylamide gel. The corresponding *K_d(app)_* is shown below each gel along with the DNA sequence of the top strand. Three independent replicates were performed for each oligonucleotide, except for ProU-40, which was done once with MotB and once with MotB-His.

To examine the DNA-binding properties of MotB in detail, we tested the ability of MotB (or MotB-His) to bind different dsDNA oligonucleotides: (1) a 37 bp fragment containing positions −29 to +8 of the promoter (Pl*_8_*) for the T4 late gene *8*, whose expression decreased 2.1-fold in the *motB^am^* infection 5 min post-infection as detailed below (dsP8, Figure 3a); (2) a 40 bp fragment containing positions +8 to +47 of the *E. coli proU* operon (ProU-40, Figure 3c); and (3) a random 32 bp oligonucleotide that is not present in either the host or viral genome (Oligo-32, Figure 3d). All three ds oligonucleotides were retarded in EMSAs, with similar *K_d(app)_*’s of 0.04 to 0.1 µM. It should be noted a comparison of MotB vs. MotB-His binding (Figure 3a,c) indicated that both proteins bind with similar affinity.

To assess whether fragment length affected MotB binding, we compared the affinity of MotB-His for ds oligonucleotides of various sizes containing the Pl*_8_* promoter motif (Figure S4a): 37 bp (Figure 3a), 23 bp (Figure S4b), and 20 bp (Figure S4c). While the 37 bp and 23 bp fragments yielded similar *K_d(app)_*’s (0.1 ± 0.03 µM and 0.09 ± 0.03 µM, respectively), MotB affinity for the 20 bp fragment decreased ~10-fold, suggesting that a fragment size greater than ~20 bp is needed for optimal binding. Finally, we also tested the ability of MotB-His to bind ssDNA, using the 37 nucleotides top strand of the ds P8 fragment (Figure 3b). In this case, affinity decreased ~6-fold, indicating a MotB preference for ds DNA.

### 3.4. DNase I Footprints Indicate that MotB Can Bind Nonspecifically Along the DNA

**Figure 4:**
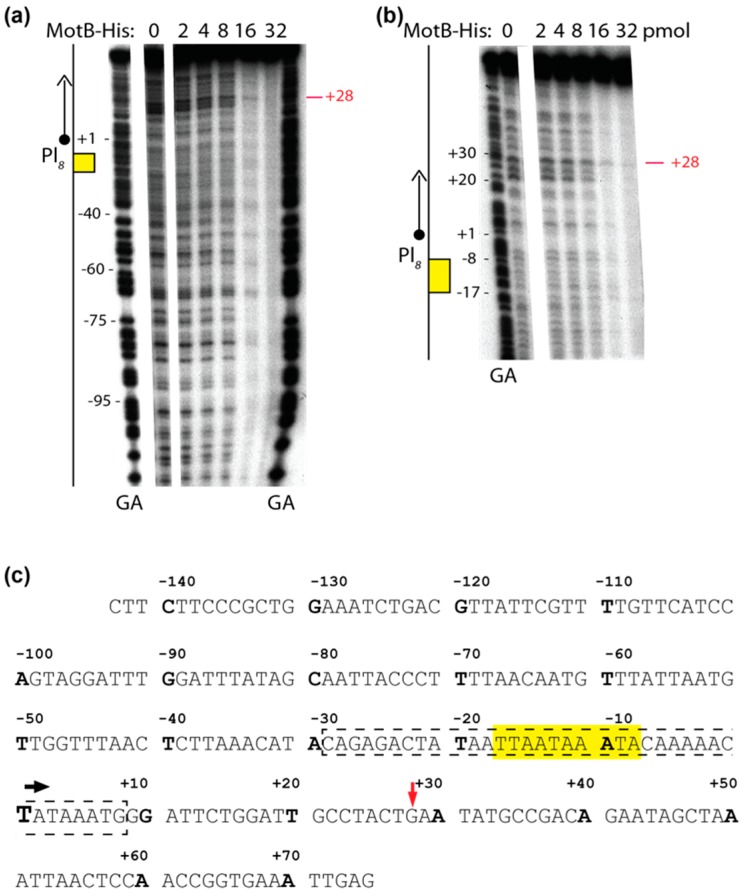
DNase I footprint of MotB on Pl*_8_* promoter DNA. The 5’-^32^P labeled DNA surrounding the Pl*_8_* promoter (positions −143 to +75; 0.05 pmol DNA (10.9 pmol total bp), top labeled) was incubated with the indicated amount of MotB-His and treated with DNase I before electrophoresis on a 5% (*w*/*v*) polyacrylamide, 7 M urea denaturing gel. Footprints in (**a**,**b**) contain the same samples; however, for (**b**) samples were electrophoresed longer to better resolve the downstream region of the DNA. GA lanes represent G + A ladder. To the left of each gel, a schematic of the Pl*_8_* promoter region is shown with the T4 late promoter TATA box depicted in yellow and the +1-transcriptional start site indicated by the circular arrow head. Footprints are representative of two independent replicates. Nontemplate sequence (**c**) of the Pl*_8_* promoter (−143 to +75) with the TATA box highlighted in yellow, the 37 bp P8 oligo used for gel shift assays indicated by the dashed box, the +1-transcriptional start site indicated by the black arrow and larger font, and the +28 position indicated by the red arrow.

To further investigate DNA specificity, we performed DNase I footprinting, using an unmodified 218 bp DNA fragment containing the sequence surrounding Pl*_8_* from positions −143 to +75 (T4 map units 85,671 to 85,888; Figure 4a,b). Thus, this region included the sequences of the P8 oligonucleotides used for EMSAs (Figure 4c). No significant protection was seen until a MotB:bp DNA ratio of ~1.5:1 (MotB at 16 pmol), when there was significant protection throughout the fragment. At a 2-fold higher concentration of MotB (32 pmol), the DNA was essentially fully protected with no detectable cleavage except for a faint band corresponding to cleavage at position +28. This result suggests that there is no preferred sequence binding motif for MotB within this fragment and that once the level of MotB relative to the DNA is high enough, MotB binding “spreads” along the entire region of the fragment.

### 3.5. H-NS and StpA Copurify with MotB

**Figure 5:**
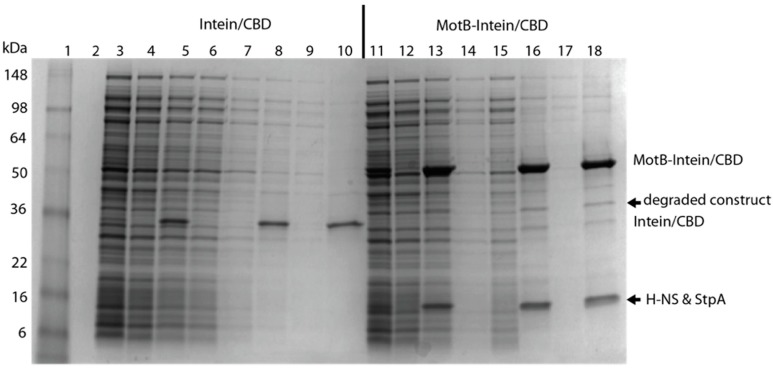
MotB copurifies with H-NS and StpA. An SDS-PAGE gel, stained with Coomassie, is shown for the following samples obtained from BL21(DE3)/pLysE containing either the vector pTXB1 (lanes 3–10) or pTXB1-MotB (lanes 11–18): lysate applied to chitin resin (lanes 3 and 11); column flow through (lanes 4 and 12); resin sample after flow through (lanes 5 and 13); first and second column wash with buffer containing 500 mM NaCl (lanes 6, 7 and 14, 15); resin sample after second 500 mM salt wash (lanes 8 and 16); column wash with buffer containing 1 M NaCl (lanes 9 and 17); final resin sample after 1 M salt wash (lanes 10 and 18). Lane 1 corresponds to SeeBlue Plus2 protein standard; corresponding molecular weights are shown on the left. Bands corresponding to MotB-Intein/CBD and Intein/CBD are indicated. Species indicated with arrows were identified by mass spectrometry. The ~36 kDa protein labeled as “degraded construct” was identified as partially degraded MotB-Intein/CBD. The ~16 kDa protein was identified as H-NS and StpA.

To determine if MotB interacts with host proteins, pull-down assays were performed using a lysate from cells producing the MotB-Intein/CBD. To control for host proteins that might interact with the Intein/CBD tag, we used cells producing only Intein/CBD as a negative control. Cell lysates were applied to chitin resin and extensively washed with high salt buffer. Resin aliquots were then removed, and proteins were detected by SDS-PAGE. For the control, the major species detected was Intein/CBD protein (Figure 5, lane 10). As expected for the MotB-Intein/CBD construct, a large amount of MotB-Intein/CBD and a small amount of Intein/CBD were detected with the lysate (Figure 5, lane 18). However, in addition, two other novel species, indicated by the black arrows, were observed. Mass spectrometry indicated that the faint, higher molecular weight band contained MotB and Intein, consistent with partial degradation of our target protein and tag. The much more intense, lower molecular weight band was determined to contain the bacterial proteins H-NS and StpA with 91% and 70% sequence coverage, respectively. H-NS was approximately 6 times more abundant in the sample, which is consistent with significantly lower level of StpA compared to H-NS in *E. coli* [39]. Because MotB-Intein/CBD was not denatured before application to the chitin column, DNA was also present as shown in Figure 2. We conclude that MotB interacts with H-NS (and StpA) either directly or indirectly through the presence of DNA.

### 3.6. MotB Interacts with Both Unmodified and Modified GHme-C DNA

**Figure 6:**
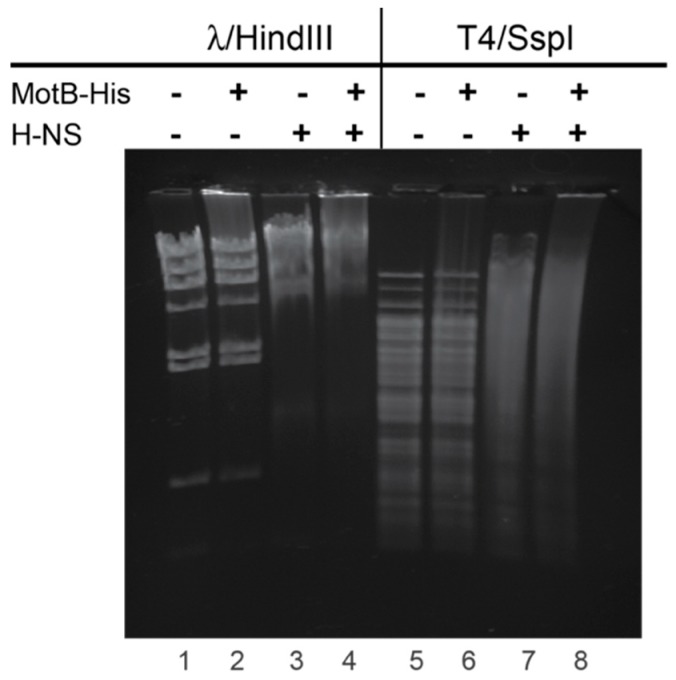
MotB and H-NS bind to both unmodified λ and GHme-C modified T4 DNA. Agarose gel shows the DNA (500 ng) λ or T4 DNA pretreated with HindIII and SspI restriction nucleases (lanes 1 and 5, respectively), after incubation with 60 pmol MotB (lanes 2 and 6), 60 pmol H-NS (lanes 3 and 7), or both (lanes 4 and 8). DNA was visualized by ethidium bromide staining and UV illumination.

Because MotB was purified from *E. coli* in the absence of a T4 infection, we deduced that the copurifying DNA present before denaturation did not contain the modification found on T4 DNA, GHme-C. To investigate if MotB could bind to the modified DNA, we performed EMSAs with T4 wt DNA, pretreated with SspI restriction enzyme, as well as unmodified lambda DNA pretreated with HindIII restriction enzyme as a control. Both DNAs were retarded by the presence of MotB, indicating that the protein binds both phage modified and unmodified DNA (Figure 6). In both cases, some DNA was severely retarded, with a fraction that barely entered the gel.

As expected, addition of H-NS to unmodified λ/HindIII DNA resulted in retardation of all the DNA fragments consistent with H-NS binding nonspecifically to DNA (Figure 6) [40]. Strikingly, the resolution of specific DNA fragments of T4/SspI is completely lost. Previous work has shown that H-NS can bind to T4 GT7 DNA, which replaces GHme-C with unmodified cytosine [41]. To our knowledge, this is the first study that has shown that H-NS can bind to GHme-C modified T4 DNA (Figure 6). Furthermore, addition of both MotB and H-NS together resulted in a more complete shift of either the λ/HindIII or T4/SspI DNA. At this point, we cannot distinguish whether H-NS and MotB bind to separate populations of DNA or bind concurrently to the same DNA. However, taking into account the length of DNA, it is conceivable that both MotB and H-NS could bind to the same fragment.

### 3.7. T4 motB^am^ Reduces Burst Size and Overexpression of motB Increases Plaque Size

**Figure 7:**
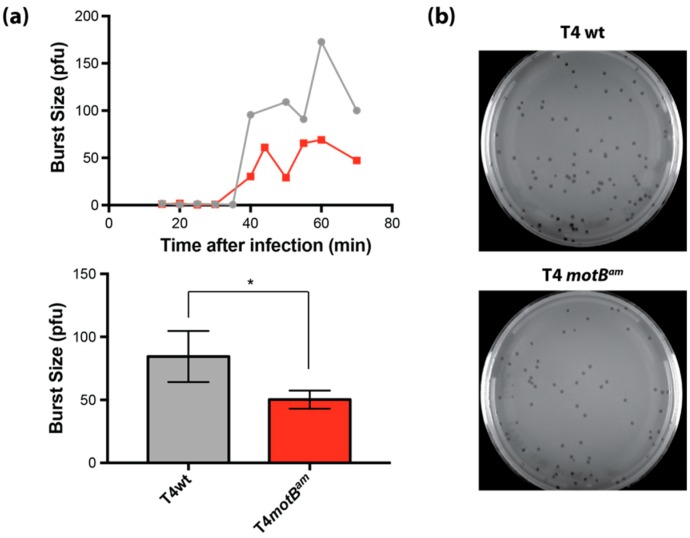
MotB improves the burst size of a T4 infection, but has no obvious effect on plaque size. (**a**) A *motB* knock-down (T4 *motB^am^*, red) reduces burst size approximately twofold compared to a wild-type infection (T4 wt, grey). Top panel shows burst size as determined by plaque forming units (pfu) over the time course of a T4 infection for a representative set of infections. Bottom panel shows a bar graph of the average burst size with standard deviations determined for three independent replicates. The asterisk (*) indicates significance as determined by an unpaired *t*-test with a *p* value ≤ 0.05. (**b**) A *motB* knock down (*motB^am^*) does not affect plaque size. Representative image of plaques formed using T4 wt (top panel) or T4 *motB^am^* (bottom panel). Images are representative of at least three independent replicates.

A T4 *motB^am^* mutant was constructed to investigate the functional phenotype of *motB* during T4 infection of non-suppressing *E. coli*. We observed ~2-fold reduction in burst size for the T4 *motB^am^* infection compared to T4 wt (Figure 7a), indicating that *motB* contributes to phage fitness although no difference in plaque size was observed between T4 wt and T4 *motB^am^* (Figure 7b). To ask whether an excess of MotB would improve phage growth, we infected cells expressing *motB* from a plasmid. Because of toxicity, synthesis of MotB was induced by the addition of 0.1% (*w*/*v*) arabinose for only 20 min before plating on LB agar containing no arabinose, thereby producing a low level of MotB prior to infection. In fact, the level of produced MotB was too low to be detected by SDS-PAGE. Nevertheless, prior production of MotB resulted in larger plaques when infected with T4 wt, T4 *motB^am^*, or T4 *motA^am^* (Figure S5), which normally yields very tiny plaques since MotA is required for Pm activation ([22,34,35], reviewed in [5]).

### 3.8. motB Knockdown has Only Mild Effects on T4 RNA Levels

Previous work had suggested that *motB* might be needed for optimal expression of certain middle genes [16] and that the MotB protein could be isolated from T4 infected cells in a complex containing DNA, RNAP, and T4-encoded factors that associate with RNAP [18]. Thus, it seemed plausible that MotB might function as a factor needed for optimal T4 gene expression. To investigate this, we performed RNA-seq and RT-qPCR analyses on RNA isolated from T4 wt and T4 *motB^am^* infections of non-suppressing *E. coli* 5 min post-infection. In a T4 wt infection, the 5 min time point corresponds to the peak of middle transcription, the end of early transcription, and the beginning of late transcription.

The RNA-seq data revealed that the expression of only six late genes, which decreased from 2 to 4.8-fold, were significantly affected in the T4 *motB^am^* infection relative to T4 wt (Table S1, Table 1) at 5 min post-infection. The expression of early and middle genes did not change. Five of the late genes were subsequently validated by RT-qPCR: *7*, *8*, *15*, *20* and *27* (Table 1). These genes encode proteins that contribute to the phage base plate, head, or tail.

To investigate this further, we observed transcripts from selected Pe’s and Pm’s as well as the specific promoters upstream of differentially expressed late genes by primer extension of RNA isolated at 1 min, 5 min, and 10 min post-infection. At 1 min post-infection, only Pe’s are significantly active; at 5 min, early transcription is waning, Pm’s are very active, and late transcription is beginning; at 10 min, most transcription is from Pl’s [42]. Primer extension analyses of Pe_73_, Pe_72.6_, and Pe_144.6_ for RNA isolated at 1 or 5 min agreed with the conclusion that Pe activity was not significantly affected (i.e., changed by more than twofold) by the *motB* knock-down (Figures S6a and S6b). Although the level of Pm RNA at 1 min was too low for analysis, primer extension products from 5 min middle RNAs were easily detected, and as expected from our RNA-seq analysis, transcript levels from the tested promoters were not significantly changed in the T4 *motB^am^* infection (Figure S6B).

**Figure 8:**
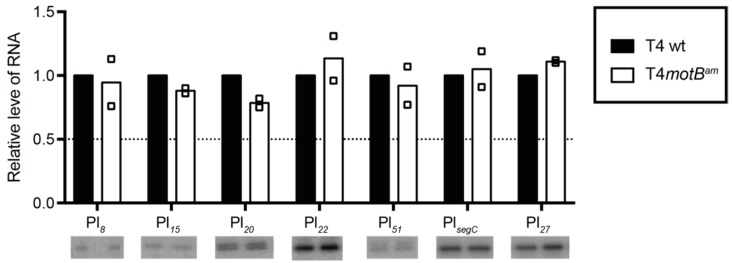
Primer extension products from selected T4 late promoters are similar in T4 wt or a T4 *motB^am^* infection at 10 min post-infection. Histograms show the level of primer extension product obtained from the indicated late promoter using RNA from a T4 *motB^am^* infection (open bar) or a T4 wt (black bar) infection; representative slices of DNA gels are shown below. Primer extensions were repeated using 2 biological replicates. The relative level of product for each replicate is shown as an open square (T4 *motB^am^*). The dotted line indicates the threshold for a twofold change in the level of RNA.

Using the 5 min RNA, we were unable to quantify the levels of all the differentially expressed late RNAs because the level of late transcription at this time is so low. However, with Pl*_22_* and Pl*_27_*, we observed that the *motB* mutation did not significantly affect transcription. At 10 min post-infection, when late transcription is robust, products derived from late promoters were easily detected, and there was no difference between levels of RNA from T4 wt or the T4 *motB^am^* infections for all the Pl’s tested (Figure 8). In particular, the levels of primer extension products from the promoters upstream of the late genes whose RNA was lower in the RNA-seq and RT-qPCR analyses (Pl*_8_*, Pl*_15_*, Pl*_20_*, Pl*_22_*, and Pl*_27_*) were unchanged. We conclude that transcription of late genes *7*, *8*, *15*, *20* and *27*, which is lower at 5 min in the T4 *motB^am^* infection, is fully restored by 10 min.

Finally, we used primer extensions within a specific early, middle, or late region at 1, 5 or 10 min post-infection, respectively, to investigate whether the absence of *motB* altered the overall transcription pattern. As seen in Figure S7, the detected 5’ ends within these regions are identical, again indicating that the presence of MotB does not affect the steady-state levels of these early, middle, and late transcripts at the times when they are highly expressed. Taken together, we conclude that MotB is not needed for normal levels of T4 transcription.

## 4. Discussion

Although many aspects of bacteriophage T4 biology have been extensively characterized, dozens of T4’s ~300 genes encode proteins whose specific functions remain unknown. Most of these are nonessential genes that are expressed early, leading to the presumption that they are helpful for host takeover, particularly under certain conditions. In this study, we have characterized one such early gene, *motB*, located in a large dispensable region of the T4 genome, called Δ12 (from *69* to *39*; map units 16,280 to 3778) [17]. Other genes located in Δ12 have been shown to be important for phage fitness under certain environmental conditions or in certain host strains and/or to be toxic when expressed in *E. coli* [17,43]. The toxicity of *motB* when it is heterologously expressed in *E. coli* and the phage burst increase in its presence lead to the conclusion that MotB is needed for optimal phage growth, perhaps through a host takeover function.

Previous work indicated that MotB can be isolated in a pre-replicative complex containing DNA, RNAP, and other polymerase-associated and DNA-binding proteins [18]. Other work reported that the expression of particular middle genes was enhanced by the presence of *motB* in a *motA* knockdown infection [16]. Taken together, these results suggested that MotB might be needed for optimal middle gene expression, hence the name, modifier of transcription B. However, our transcriptome analyses indicate that at 5 min after infection, when middle gene expression is robust, neither early nor middle gene RNA levels are affected in a T4 *motB^am^* infection. Rather only the expression of a few late genes is lowered at 5 min post-infection. By 10 min when late transcripts are highly expressed, transcription of these genes has recovered. Therefore, it seems unlikely then that MotB directly regulates T4 gene expression unless this function is redundant within the genome.

Our biochemical analyses indicate that MotB is a protein that binds to both unmodified and T4 wt GHme-modified DNA. It binds very tightly to host DNA when produced heterologously in vivo, it binds with ~100 nanomolar affinity to various ds oligonucleotides, and in EMSAs it retards each of the λ/HindIII fragments. The DNase I footprint of MotB on an unmodified 218 bp fragment, which includes the T4 late promoter P8, does not reveal a high affinity binding site despite the down-regulation of late gene 8 in the T4 *motB^am^* infection. Instead, we observe MotB protection of the entire region of the DNA once a sufficient protein concentration is reached. This type of binding profile is indicative of a protein that can “spread” along the DNA with little, if any sequence specificity, perhaps through protein–protein cooperativity. However, we cannot eliminate the possibility that MotB has a high affinity site that was not present in the tested DNAs.

Curiously, MotB copurifies with H-NS and StpA, *E. coli* histone-like proteins that also can spread along the DNA with limited DNA specificity. H-NS, and its less abundant homolog StpA, target AT-rich DNA sequences, condensing genomic DNA through the formation of ordered and looped structures, and typically repress transcription at the affected region (reviewed in [8]). Our gel retardation assays indicate that H-NS binds to T4 wt GHMe-modified DNA and that the retardation of either unmodified or GHMe-modified DNA increases in the presence of both MotB and H-NS over that observed with either alone (Figure 6). At this point it is unclear whether both H-NS and MotB bind to the same region of DNA simultaneously. Furthermore, despite multiple efforts to show a direct interaction between H-NS and MotB, we have not observed an interaction in the absence of DNA.

Given the high predicted pKa of MotB (9.23) and its presence in the T4 dispensable region Δ12, we can determine its relative abundance during infection from previous 2-D gels of radiolabeled T4 proteins at various times after infection [44]. We identify a highly basic protein, which migrates as predicted for MotB’s size and predicted pKa and is missing in the T4 Δ12 infection. The level of this protein is high when T4 proteins are labeled from 1 to 3 min post-infection and remains high even with 7–9 min post-infection labeling. This identification is supported by another finding that MotB (designated as p17.6) production initiates early after infection (0–4 min) and continues as infection progresses (7–12 min) [18]. It is known that T4 factors begin to degrade the host DNA shortly after infection, but degradation of host DNA to acid-soluble products is still less than 50% at 15 min post-infection [45]. It seems reasonable then to conclude that the high levels of MotB would be able to bind host and/or T4 DNA throughout the time it is present. Given the copurification of MotB with H-NS and StpA, two proteins that would be present on the host DNA at the time of infection and could interact with T4 wt DNA, it is tempting to speculate that MotB binding to the DNA might work to counteract H-NS/StpA binding. However, additional work is needed to determine the localization of MotB and H-NS relative to host and phage DNA. Nonetheless, it is clear that heterologous expression of *motB* is highly toxic, resulting in rapid cell lysis of BL21(DE3) cells and growth arrest of TOP10F’ cells. The bactericidal properties of phage proteins when expressed in their host is one of the driving forces of phage research. The mechanisms by which proteins such as MotB inhibit bacterial growth could provide insight for the development of future antibiotics.

## Figures and Tables

**Table 1:** A T4 *motB* knock-down lowers some T4 late gene expression at 5 min post-infection. RNA-seq was performed for three independent T4 wt and T4 *motB^am^* infections of NapIV NS *E. coli.* Differential expression was determined using the Baggerley’s Test in CLC Genomics Workbench with FDR corrected *p* values. Genes with ≥ 2-fold decreasing expression and a *p* value ≤ 0.05 are listed. For RT-qPCR, genes *frd.2, nrdD,* and *motA*, which were unaffected in the RNA-seq data, were used as endogenous controls.

			RNA-SEQ		RT-QPCR
Gene Type	Gene	Function	Fold Change	Corrected *p* Value	Relative Expression (±SD)
Late	*7*	Base plate wedge component	−2.0	1.04 × 10^−40^	−2.5 (±0.4)
Late	*15*	Proximal tail sheath stabilizer, connector	−4.8	1.72 × 10^−7^	−2.5 (±0.5)
Late	*27*	Base plate hub subunit	−3.4	1.87 × 10^−6^	−2.7 (±0.9)
Late	*20*	Portal vertex protein of the head	−2.1	8.16 × 10^−6^	−1.9 (±0.4)
Late	*8*	Base plate hub subunit	−2.1	0.0002	−2.0 (±0.3)
Late	*22* *	Prohead core protein; precursor to internal peptides	−3.3	1.98 × 10^−7^	−1.3 (0.2)

* Gene 22 was determined to be a false positive.

## Supplementary Materials

The following are available online at http://www.mdpi.com/1999-4915/10/7/343/s1, Figure S1: GC-contents of total RNA have a bimodal distribution for T4 wt infections; Figure S2: Toxicity of C-terminally tagged MotB constructs in *E. coli*; Figure S3: Purification of MotB; Figure S4: MotB binding to P8 oligonucleotides of various lengths; Figure S5: Effect of heterologous production of MotB on T4 plaque size; Figure S6: Primer extension products from selected T4 promoters in a T4 wt or a T4 *motB^am^* infection at 1 min and 5 min post-infection; Figure S7: Transcripts in early, middle, and late regions are similar in T4 wt (+) or T4 *motB^am^* (*am*) infections of NapIV NS; and Table S1: Summary of all RNA-seq and RT-qPCR data.
