# Supplementary material for: The Bacteriophage T4 MotB Protein, a DNA-Binding Protein, Improves Phage Fitness
Source: Viruses. 2018 Jun 26;10(7):343. doi: 10.3390/v10070343 (PMC6070864; doi:10.3390/v10070343)
Supplement: Supplementary file 1 [file viruses-10-00343-s001.zip › MotB_viruses_supplemental_revisions1_final/MotB_viruses_supplemental_revisions1_final.docx]

**Supplemental Materials**

**
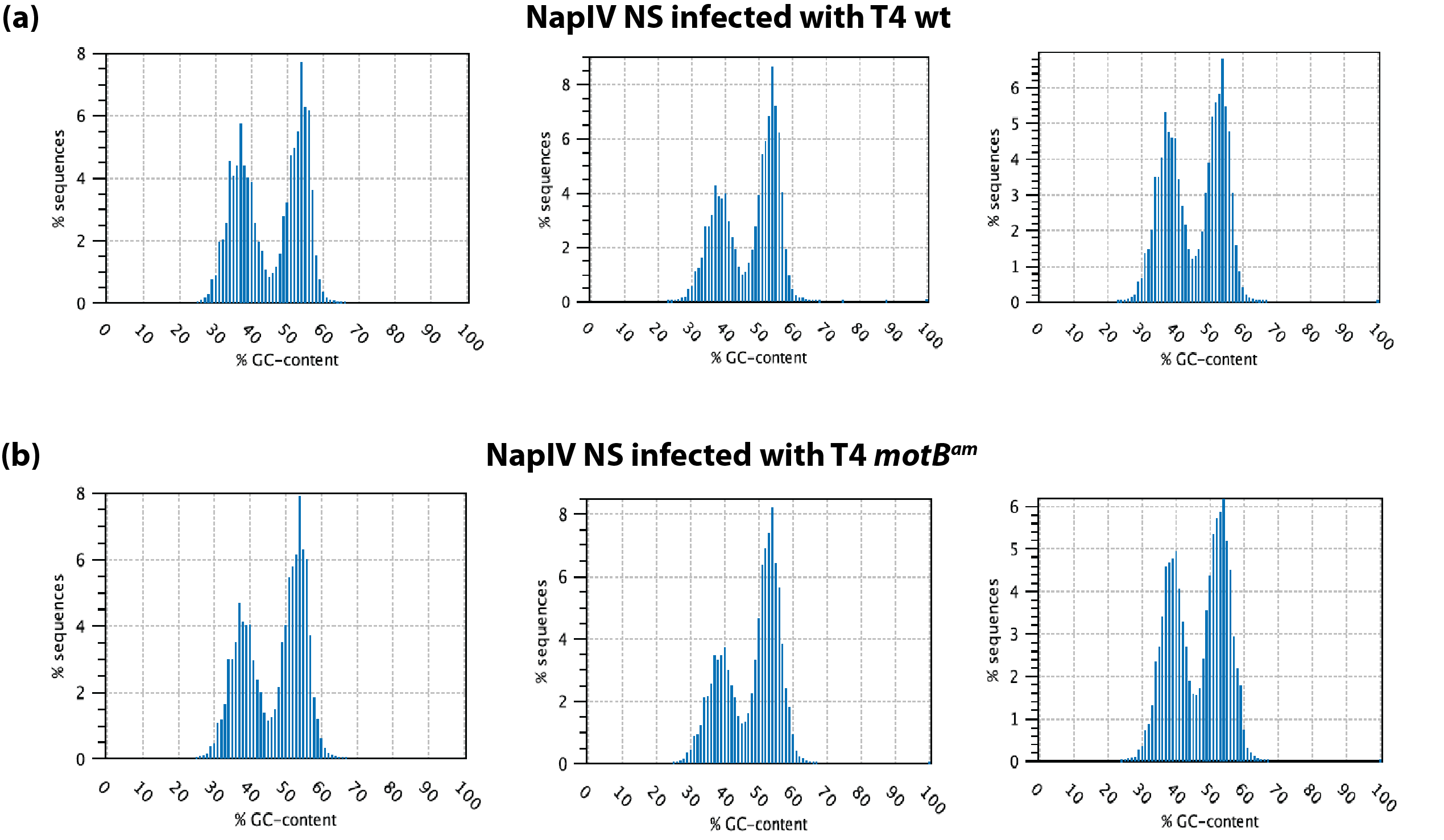
**

**Figure S1. GC-contents of total RNA have a bimodal distribution for T4 infections.** The %GC-content of total reads from RNA-seq for each replicate of (**a**) T4 wt and (**b**) T4 *motB^am^* infections of NapIV NS *E. coli* was determined during quality analysis of sequence data in CLC Genomics Workbench. A bimodal distribution is consistent with a mixed population of host-encoded (~55% GC) and T4-encoded (34.5% GC) RNA.

**
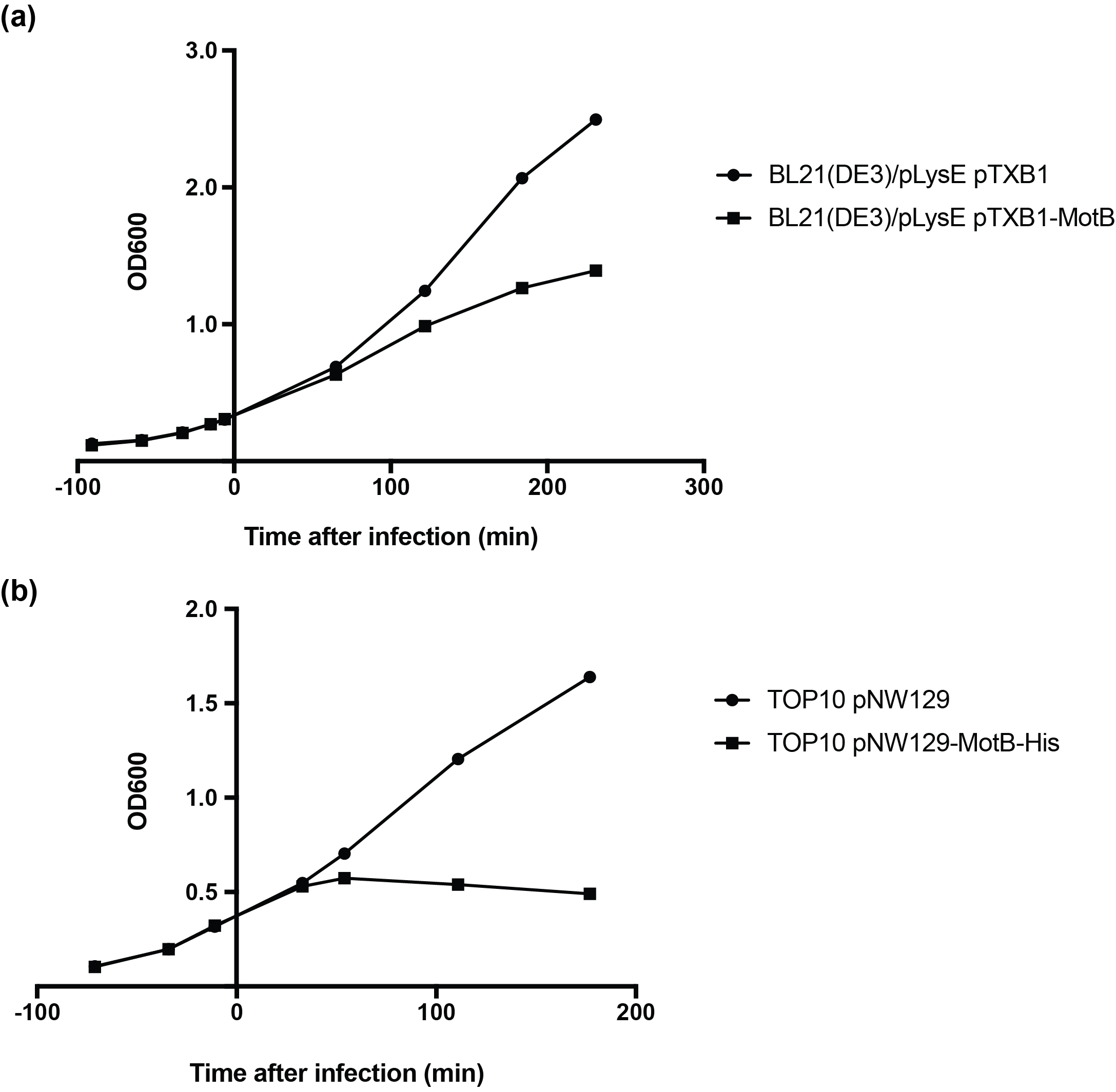
**

**Figure S2. Toxicity of C-terminally tagged MotB constructs in *E. coli.*** (**a**) BL21(DE3)/pLysE containing either the control vector pTXB1(circles) or expression vector pTXB1-MotB (squares) was grown at 25°C, and protein production was induced by the addition of 2 mM IPTG. (**b**) TOP10F’ containing either the empty vector pNW129 (circles) or expression vector pNW129-MotB (squares) was grown at 37°C, and protein production was induced by the addition of 0.2% (w/v) arabinose. Growth curves are representative of at least three independent replicates.


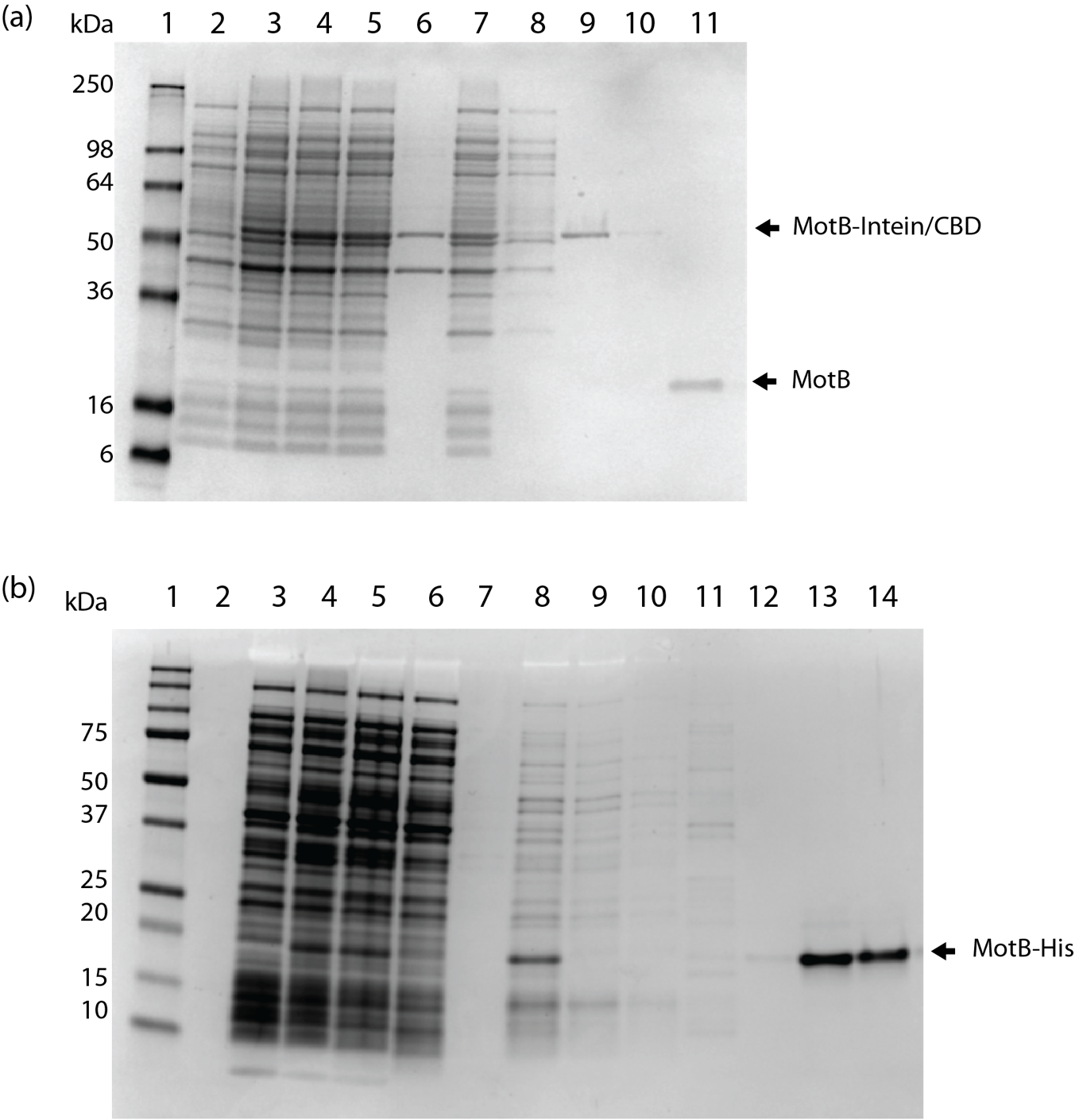


**Figure S3. Purification of MotB.** SDS-PAGE gels, stained with Coomassie, of fractions from the purification of MotB (from MotB-Intein/CBD) (**a**) and MotB-His (**b**). Fractions in (**a**) are pre-induction (lane 2), 4 hrs post-induction negative control (lane 3), 4 hrs post-induction sample (lane 4), supernatant (lane 5), pellet (lane 6), chitin column flow through (lane 7), chitin column wash (lane 8), resin sample at 0 hr (lane 9), resin sample at 42 hrs after elution (lane 10), and elution (lane 11). Fractions in (**b**) are pre-induction (lane 3), post-induction (lane 4), sonication (lane 5), supernatant 1 (lane 6), supernatant 2 (lane 7), resuspended pellet (lane 8), Ni^2+^ column flow through (lane 9), binding buffer (lane 10), wash buffer (lane 11), and elution (lane 12-14). The protein standard in lane 1 of (**a**) and (**b**) is SeeBlue Plus2 Protein Standard (Invitrogen) and Precision Plus Protein Standard All Blue (Bio-Rad), respectively.


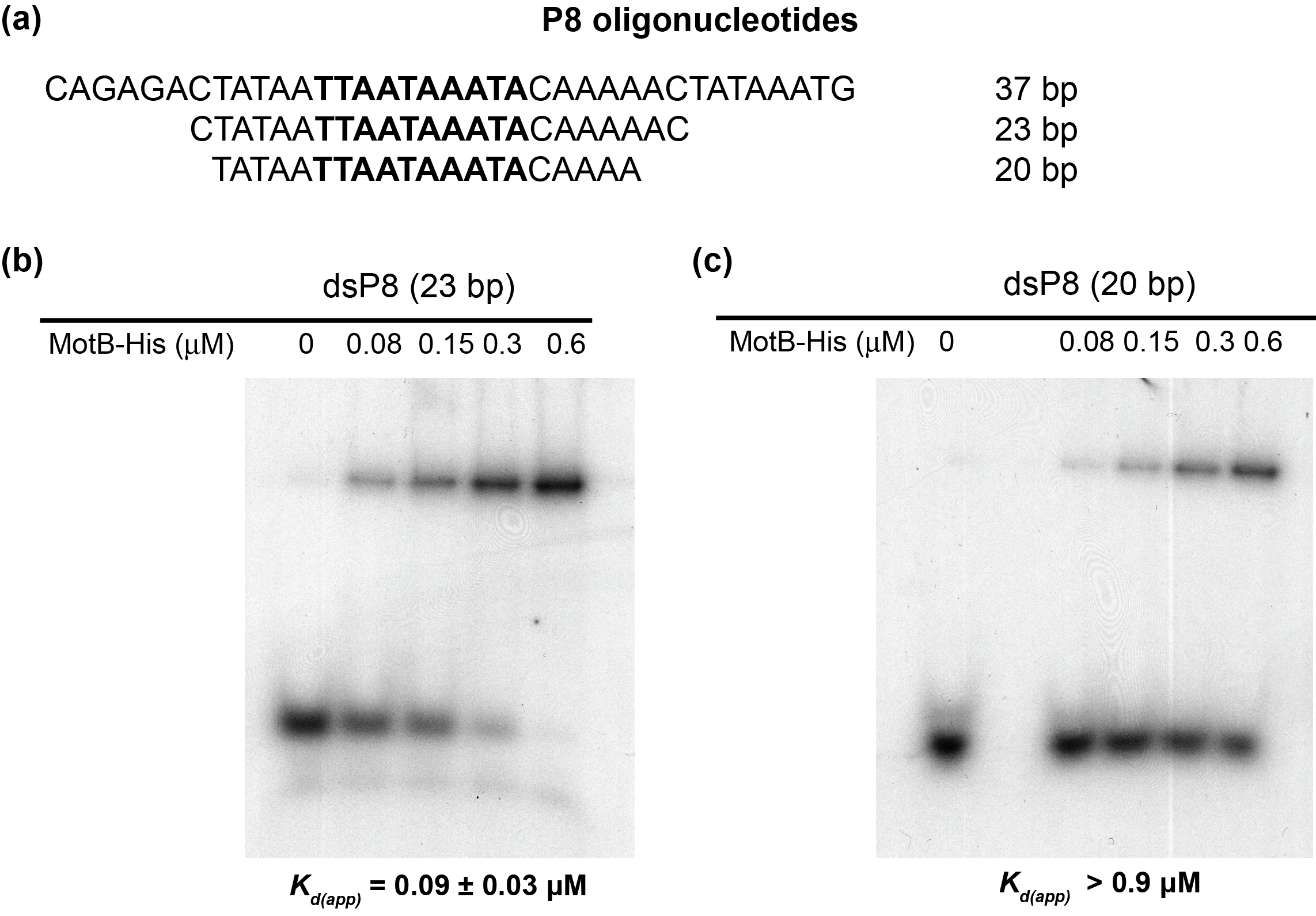


**Figure S4. MotB binding to P8 oligonucleotides of various lengths.** (**a)** Sequences of the top strand of the ds P8 oligonucleotides used in this study. Representative EMSA gels for MotB-His binding to (**b**) the 23 bp and (**c**) the 20 bp P8 oligonucleotides are shown. (EMSA gel for the 37 bp P8 oligonucleotide is in Figure 3a.) DNA (1 nM) was incubated with the indicated concentrations of MotB-His at 37°C for 10 min and then electrophoresed on a 12% native gel. The corresponding *K_d(app)_* is shown below each gel. Three and two independent replicates were performed for the 23 bp and 20 bp fragments, respectively.

##
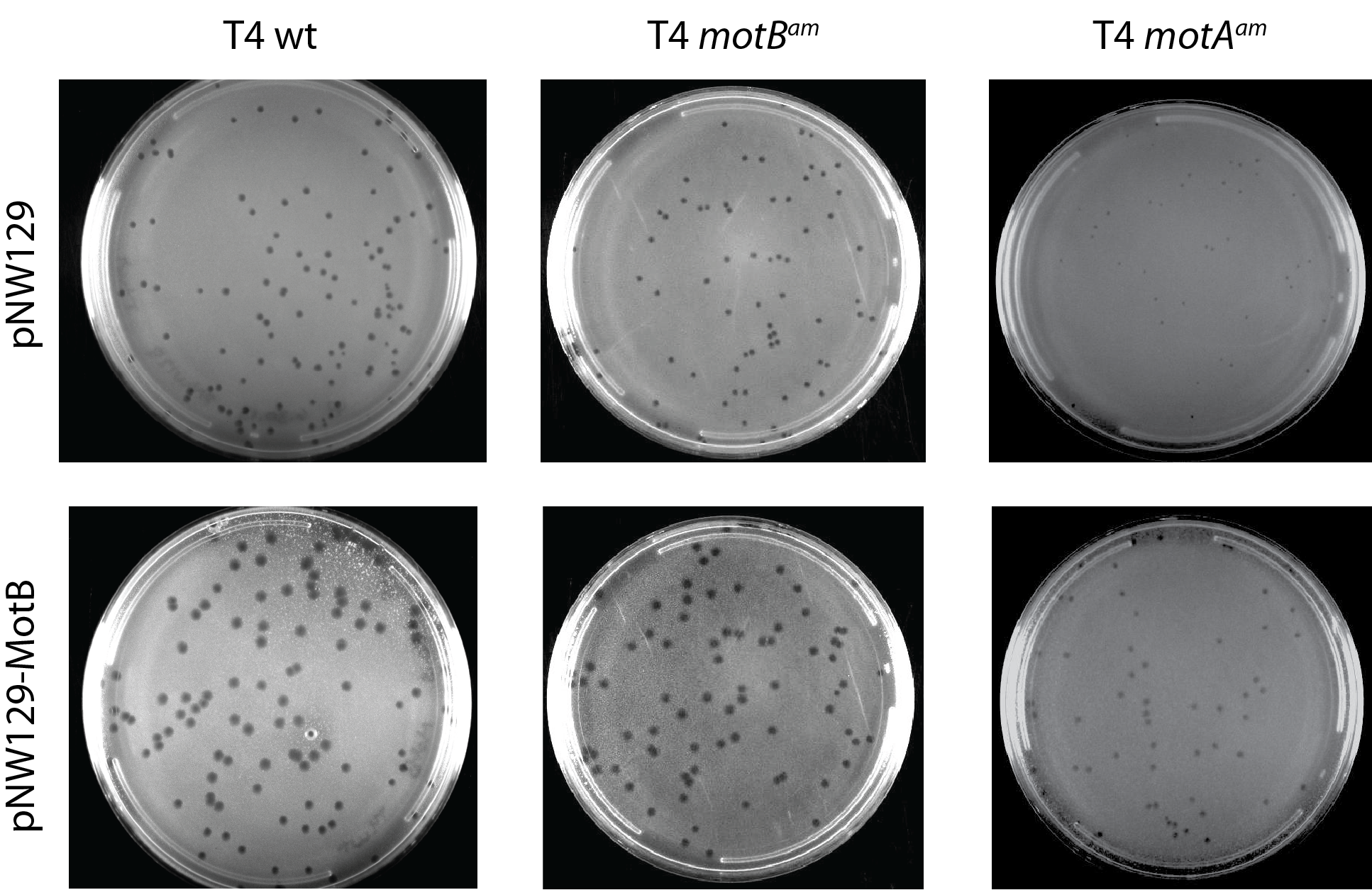


**Figure S5. Effect of** heterologous production of MotB **on T4 plaque size.** Low levels of MotB produced prior to infection increase T4 plaque size. BL21(DE3) containing either empty vector (pNW129, top panels) or expression vector (pNW129-MotB, bottom panels) was grown until early exponential phase and then induced with 0.1% (w/v) arabinose for 20 min prior to infection by T4 wt (left panels), T4 *motB^am^* (center panels), or T4 *motA^am^* (right panels). Images are representative of at least three independent replicates.

**
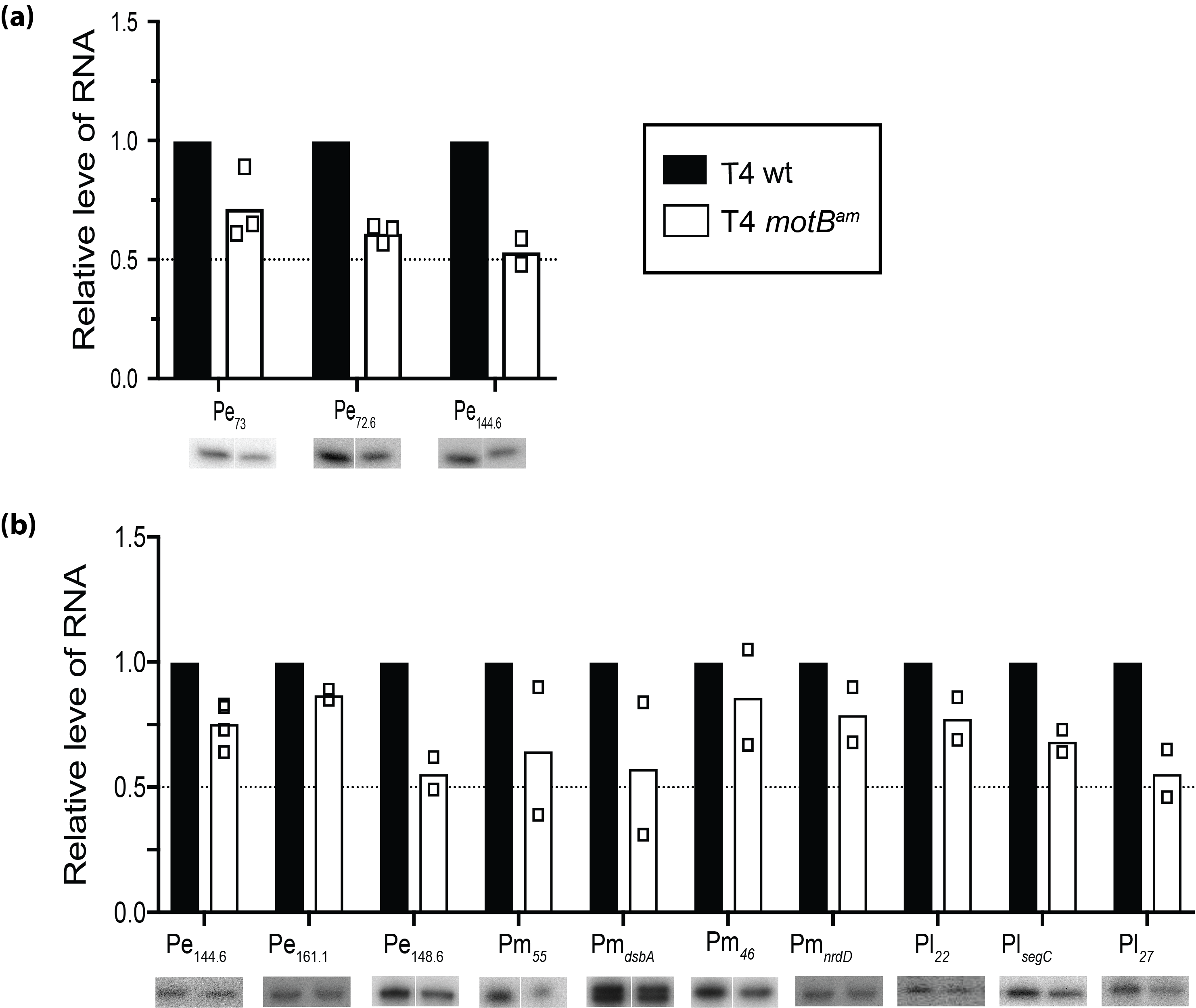
**

**Figure S6. Primer extension products from selected T4 promoters in a T4 wt or a T4 *motB^am^* infection at 1 min and 5 min post-infection.** Histograms show the level of primer extension product obtained from the indicated promoters using RNA from a T4 *motB^am^* infection (open bar) relative to that from a T4 wt infection (black bar) at 1 min (**a**) and 5 min (**b**) post-infection; representative slices of DNA gels are shown below. Primer extensions were repeated using 2 biological replicates. The relative level of product for each replicate is shown as an open square (T4 *motB^am^*). The dotted line indicates the threshold for a 2-fold change in the level of RNA. Primer extensions were also performed for Pm*_46_*, Pm*_dsbA_*, and Pm*_55_* at 1 min post-infection and Pe_72.6_, Pe_73_, Pl*_8_*, Pl*_20_*, and Pl*_51_* at 5 min post-infection_,_ however, the signal was too low to quantify accurately.


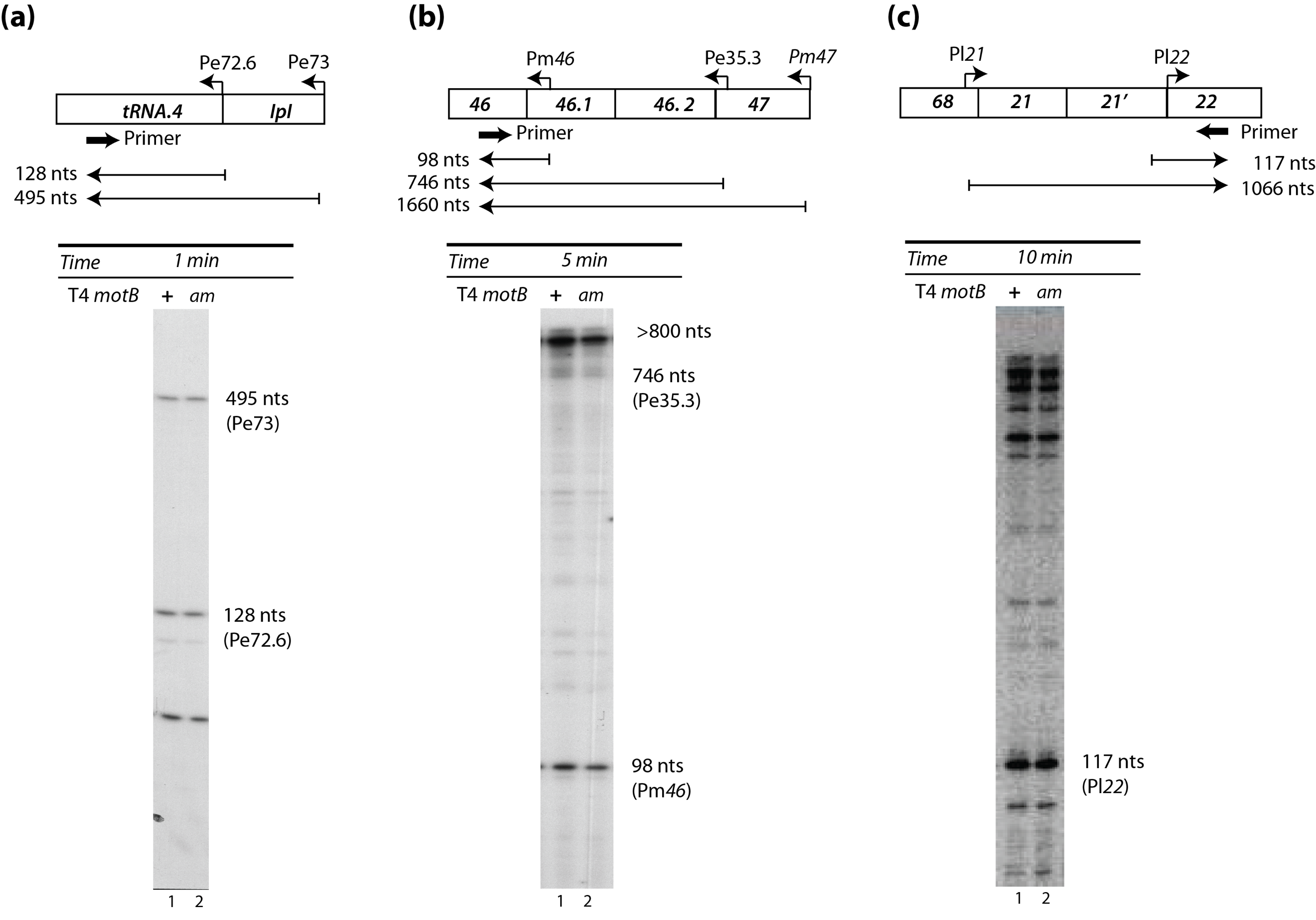


**Figure S7. Transcripts in early, middle, and late regions are similar in T4 wt (+) or T4 *motB^am^* (*am*) infections of NapIV NS.** Representative gels show primer extension products from primers in the early gene *tRNA.4* at 1 minute (**a**), middle gene *46* at 5 minutes (**b**) and late gene 55 at 10 minutes (**d**) post-infection. Primer extensions were repeated using 2 biological replicates. Above each gel is a schematic of the T4 genomic map from map units 73,329-73,903 (**a**), 33,302-36,576 (**b**), and 103,023-104,095 (**c**) showing previously identified promoters and relevant transcripts with lengths of predicted primer extension products.

**Table S1.** **Summary of all RNA-seq and RT-qPCR data.** Genes with no significant change in expression are in white, whereas genes that had a ≥ 2-fold decrease in expression with a *p* value ≤ 0.05 are in green. Values for genes whose changes in gene expression were verified by RT-qPCR are given. Fold changes that were not consistent between RNA-seq and RT-qPCR are given in red. (excel file)
